# Identifying the best predictive diagnostic criteria for psoriasis in children (< 18 years): a UK multicentre case–control diagnostic accuracy study (DIPSOC study)*

**DOI:** 10.1111/bjd.20689

**Published:** 2021-11-24

**Authors:** E. Burden‐Teh, R. Murphy, S. Gran, T. Nijsten, C. Hughes, A. Abdul‐Wahab, A. Bewley, N. Burrows, S. Darne, J.E. Gach, R. Katugampola, C.S. Jury, K. Kuet, J. Llewellyn, T. McPherson, J.C. Ravenscroft, S. Taibjee, C. Wilkinson, K.S. Thomas

**Affiliations:** ^1^ Centre of Evidence Based Dermatology School of Medicine University of Nottingham Nottingham UK; ^2^ Department of Dermatology Nottingham University Hospitals NHS Trust Nottingham UK; ^3^ Department of Dermatology Sheffield Children’s NHS Foundation Trust Sheffield UK; ^4^ Department of Dermatology Erasmus Medical Centre Rotterdam the Netherlands; ^5^ Department of Dermatology St George’s University Hospitals NHS Foundation Trust London UK; ^6^ Department of Dermatology Barts Health NHS Trust London UK; ^7^ Department of Dermatology Cambridge University Hospitals NHS Foundation Trust Cambridge UK; ^8^ Department of Dermatology The James Cook University Hospital Middlesbrough UK; ^9^ Department of Dermatology University Hospitals Coventry and Warwickshire NHS Trust Coventry UK; ^10^ Department of Dermatology Cardiff and Vale University Health Board Cardiff UK; ^11^ Department of Dermatology Royal Hospital for Children Glasgow UK; ^12^ Department of Dermatology Oxford University Hospitals NHS Foundation Trust Oxford UK; ^13^ Department of Paediatric Dermatology Nottingham University Hospitals NHS Trust Nottingham UK; ^14^ Department of Dermatology Dorset County Hospital NHS Foundation Trust Dorchester UK; ^15^ Department of Dermatology University Hospital Plymouth NHS Trust Plymouth UK

## Abstract

**Background:**

In children, psoriasis can be challenging to diagnose. Difficulties arise from differences in the clinical presentation compared with adults.

**Objectives:**

To test the diagnostic accuracy of previously agreed consensus criteria and to develop a shortlist of the best predictive diagnostic criteria for childhood psoriasis.

**Methods:**

A case–control diagnostic accuracy study in 12 UK dermatology departments (2017–2019) assessed 18 clinical criteria using blinded trained investigators. Children (< 18 years) with dermatologist‐diagnosed psoriasis (cases, *N* = 170) or a different scaly inflammatory rash (controls, *N* = 160) were recruited. The best predictive criteria were identified using backward logistic regression, and internal validation was conducted using bootstrapping.

**Results:**

The sensitivity of the consensus‐agreed criteria and consensus scoring algorithm was 84·6%, the specificity was 65·1% and the area under the curve (AUC) was 0·75. The seven diagnostic criteria that performed best were: (i) scale and erythema in the scalp involving the hairline, (ii) scaly erythema inside the external auditory meatus, (iii) persistent well‐demarcated erythematous rash anywhere on the body, (iv) persistent erythema in the umbilicus, (v) scaly erythematous plaques on the extensor surfaces of the elbows and/or knees, (vi) well‐demarcated erythematous rash in the napkin area involving the crural fold and (vii) family history of psoriasis. The sensitivity of the best predictive model was 76·8%, with specificity 72·7% and AUC 0·84. The *c*‐statistic optimism‐adjusted shrinkage factor was 0·012.

**Conclusions:**

This study provides examination‐ and history‐based data on the clinical features of psoriasis in children and proposes seven diagnostic criteria with good discriminatory ability in secondary‐care patients. External validation is now needed.

## Graphical abstract

**What is already known about this topic?**

- A diagnosis of psoriasis may be delayed in children and young people, and psoriasis may be misdiagnosed in primary and secondary care.
- Diagnostic criteria for psoriasis in adults and children have been lacking.
- The development of criteria will aid recognition and clinical diagnosis of psoriasis, and provide a disease definition for clinical trials and epidemiological studies.
- Studies to develop diagnostic criteria should aim to minimize bias in the study design.

**What does this study add?**

- The consensus‐agreed 16 diagnostic criteria and proposed scoring system demonstrated good diagnostic accuracy.
- Using statistical modelling, a shortlist of the seven best predictive diagnostic criteria was identified. The presence of two or more of these criteria had a sensitivity and specificity of over 70%.
- The criteria provide a reminder to clinicians that psoriasis in children can often develop in skin covered by hair and clothing.

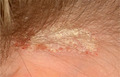

**Linked Comment:** R.J.G. Chalmers. Br J Dermatol 2022; 186:213–214.

Plain language summary available online

Psoriasis is a chronic immune‐mediated inflammatory skin disease affecting the skin and joints. The World Health Organization (WHO) has identified psoriasis as a serious noncommunicable disease and an area of unmet health need.^1^ Ensuring prompt diagnosis and identifying other priority areas for research are highlighted by both the WHO and the Psoriasis Priority Setting Partnership.^2^, ^3^

Making the diagnosis of psoriasis in children and young people can be more challenging than in adults. The presentation of psoriasis in children is often more subtle, with thinner, less hyperkeratotic plaques. The distribution often involves the flexures, face and skin covered by clothing and hair, which can be easily missed if these areas are not specifically asked about and examined.^4^, ^5^ Psoriasis in children is also under‐recognized in primary and secondary care. Reasons for this may include a lack of awareness that psoriasis can develop from infancy onwards, and psoriasis being misdiagnosed as other common childhood rashes such as atopic dermatitis/eczema, skin infections and exanthems.^6^, ^7^ The evidence to guide treatment and monitoring in childhood psoriasis is limited. For many children psoriasis can persist into adulthood and there is the potential for a cumulative negative effect over many years.^8^, ^9^, ^10^, ^11^

Currently, diagnosis is based on the recognition of clinical signs and symptoms. There are no diagnostic criteria in routine use in clinical practice or research.^12^ The lack of a standardized disease definition and case ascertainment impacts on the validity and generalizability of the evidence, and is a limitation of many existing studies.^13^, ^14^, ^15^, ^16^ Also, timely recognition of psoriasis is important for referral to a specialist, access to effective treatment and identification of juvenile psoriatic arthritis.^8^

To address this an eDelphi consensus study was completed with the International Psoriasis Council to agree a list of criteria important for the diagnosis of psoriasis in children and to propose a scoring algorithm for diagnosis.^17^ The aim of this study (DIPSOC) was to test the diagnostic accuracy of the consensus‐agreed criteria and to refine the criteria using multivariate analysis. Through refinement the aim was to identify a shortlist of the best predictive criteria.

## Patients and methods

### Protocol, ethics and study registration

The DIPSOC study protocol has been published as an open‐access manuscript.^18^ A summary of the protocol contents is provided below, highlighting any changes made as post hoc decisions. Health Regulatory Authority and National Health Service Research Ethics Committee (REC) approvals were granted in February 2017 (REC reference 17/EM/0035). The study was registered on the ISRCTN website in November 2017 (https://doi.org/10.1186/ISRCTN98851260).

### Study design and setting

DIPSOC was a multicentre diagnostic accuracy case–control study that recruited in 12 UK paediatric dermatology departments. A nested substudy following a cohort of children with possible or indeterminate psoriasis is ongoing. The study follows the STARD and TRIPOD reporting guidelines.^19^, ^20^

### Objectives

The primary objective of the DIPSOC study was to test the diagnostic accuracy of the consensus‐agreed criteria for plaque psoriasis in children and young people and to develop a shortlist of the best predictive diagnostic criteria using multivariate analysis. The secondary objectives were: (i) to compare the diagnostic performance of the consensus‐agreed diagnostic criteria and the best predictive criteria for plaque psoriasis in children and young people, (ii) to assess the interobserver variability in the diagnostic criteria assessment and (iii) to assess the variability in the reference standard for psoriasis.

### Participant selection

Inclusion criteria were children and young people aged 0–18 years with active skin disease (rash present) at the time of assessment and a dermatologist’s diagnosis made in a paediatric dermatology clinic of either (i) psoriasis (cases) or (ii) a scaly inflammatory rash other than psoriasis (controls). Children and young people with possible or indeterminate psoriasis, or pustular or erythrodermic psoriasis, or without a dermatologist’s confirmed diagnosis of their skin disease were excluded.

### Study recruitment and assessment

Consecutive new and follow‐up patients were identified in clinic or from existing medical records. Potential participants who met the eligibility criteria were approached by their usual dermatology team and recruited. The index test was divided into two parts: (i) the 16 consensus‐agreed diagnostic criteria and scoring algorithm (one major and/or three minor criteria) identified through an eDelphi consensus study with the International Psoriasis Council (Table 1), and (ii) the best predictive criteria developed in this study from 18 criteria (16 consensus criteria plus two criteria close to reaching consensus) using multivariate analysis. The reference standard was a dermatologist’s diagnosis, deemed clinically appropriate. The index test and reference standard data were obtained on the same day.

**Table 1:** Consensus‐agreed diagnostic criteria from an eDelphi study with the International Psoriasis Council.^17^ Two additional diagnostic features (*) have also been included that were close to reaching consensus and were emphasized as important in the feedback from experts

**Major criteria**
Scaly erythematous plaques on the extensor surfaces of the elbows and knees
Scaly erythematous plaques on the trunk triggered by a sore throat or other infection
Raindrop plaques typical of guttate disease on the trunk or limbs
**Minor criteria**
Scale and erythema in the scalp involving the hairline
Retroauricular erythema (including behind the earlobes)
Scaly erythema inside the external auditory meatus
Persistent well‐demarcated erythematous scaly rash anywhere on the body
Fine scaly patches involving the upper thighs and buttocks
Well‐demarcated erythematous rash in the napkin area involving the crural folds
Persistent erythema in the umbilicus
Nail pitting
Onycholysis of the nail(s)
Subungual hyperkeratosis of the nail(s)
Positive family history of psoriasis
Koebner phenomenon
Fusiform swelling of a toe or a finger suggestive of dactylitis
*Persistent well‐demarcated facial rash with fine or absent scale
*Natal cleft erythema and/or skin splitting

At the research visit, data on demographics, quality of life [Children’s Dermatology Life Quality Index (CDLQI) and Child Health Utility 9D] and the presence or absence of each of the 18 diagnostic criteria on history and examination were collected. The investigator performing the assessment of diagnostic criteria had completed standardized training and was blinded to the participant’s diagnosis. To evaluate interobserver variability in assessment of the diagnostic criteria, the assessment was conducted consecutively by two independent assessors in the first 40 participants where two assessors were available. Data on the reference standard, disease history and severity were extracted from the medical record.

### Sample size

The full statistical analysis plan was finalized before the end of recruitment and is available at: www.nottingham.ac.uk/go/dipsoc. Two calculations were made based on the two parts of the primary objective. The highest value was from the TRIPOD rule of thumb of 10 observations for each predictor variable. For 16 consensus‐agreed criteria, a sample size of 160 cases and 160 controls was required (320 participants in total).

### Data analysis

Stata version v16.0 was used to undertake the analysis (StataCorp, College Station, TX, USA). The participant characteristics of the study population were described using descriptive statistics; continuous variables that were normally distributed are presented as mean (SD) and categorical variables as number and percentage. The diagnostic accuracy of the consensus‐agreed criteria, based on the suggested scoring algorithm, was calculated as sensitivity, specificity, area under the curve (AUC) and likelihood ratio.

#### Predictive model

The frequency, sensitivity, specificity, univariate odds ratio and likelihood ratios of the individual 18 diagnostic criteria were calculated. Diagnostic criteria that did not reach 80% sensitivity and 80% specificity were included as predictors (minor criteria); this was an a priori decision. Diagnostic criteria with fewer than 10 observations were excluded because infrequently seen clinical signs would not be helpful in the majority of children; this was a post hoc decision.

The predictive model used backward logistic regression and the criteria in the final model were defined as the ‘best predictive criteria’. The linear predictor using coefficients in the model was used to estimate the probability of psoriasis. The sensitivity, specificity, AUC and likelihood ratios of the predictive model were calculated.

Multicollinearity, calibration and discrimination were assessed using cross‐tabulation, the Hosmer–Lemeshow statistic and receiver operator characteristic (ROC) curves, respectively.

The ROC curves for the consensus‐agreed diagnostic criteria and the best predictive diagnostic criteria were compared visually. The interobserver variability in the assessment of the individual diagnostic criteria was estimated using the Kappa statistic.

#### Stratification

Stratification was used to assess the diagnostic ability of the criteria in different subgroups: age (< 10 years or ≥ 10 years), sex and dermatological experience of the assessor. Other planned stratification analyses were not possible due to insufficient data in the strata leading to unstable estimates.

#### Internal validation

The bootstrap procedure was conducted for internal validation; this was repeated 1000 times to obtain a distribution of optimism estimates and the average optimism was calculated.^21^ The bootstrap‐corrected *c*‐statistic, calibration in the large and calibration slope were computed by subtracting the optimism from the original values.

#### Missing data

The proportions of missing data for each variable are presented as numbers and percentages. A complete‐case analysis (all diagnostic criteria observations recorded) was used for the predictive model, and the effect of coding missing observations as ‘yes’ or ‘no’ on the model was explored.

#### Exploration of different cutoffs

A post hoc decision was made to explore the diagnostic accuracy of setting different cutoffs of the positive best predictive criteria – for example, three or more of the best predictive criteria. This was to simulate how the criteria may be most naturally used in clinical practice, where clinicians would be interested in the diagnostic accuracy of a minimum number of diagnostic criteria.

#### Protocol amendment

Variability in the reference standard was not investigated because an insufficient number of clinical images of suitable quality were available for data collection in the study.

#### Patient and public involvement

Patient and public involvement through a patient coinvestigator and the Young Person’s Advisory Group for Research have been integral to the study question, study design and conduct of the study.

## Results

### Study population

In total 330 children and young people (< 18 years of age) were recruited between October 2017 and March 2019. Of these, 170 had a dermatologist’s confirmed diagnosis of psoriasis (cases) and 160 had been diagnosed with a different inflammatory skin disease (controls). The participant characteristics are presented in Tables 2 and 3. Cases were more often female (60·0% vs. 41·9%), were older at the time of the research visit (11·1 vs. 7·4 years) and onset of the rash (7·0 vs. 1·2 years), and were more often of white ethnicity (80·0% vs. 59·4%).

**Table 2:** Demographic characteristics of cases (psoriasis) and controls (other inflammatory skin diseases) included in the DIPSOC study

	Cases (*n* = 170)	Controls (*n* = 160)
Age of participants (years)		
Mean (SD); range	11·1 (3·6); 1·3–17·9	7·4 (5·0); 0·4–17·6
Age at diagnosis (years)		
Mean (SD); range	9·2 (3·7); 1·14–17·7	4·7 (4·2); 0–17·1
Missing	4 (2·4)	10 (6·3)
Age at onset of skin rash or symptoms (years)		
Mean (SD); range	7·0 (3·7); 0–17	1·2 (3·1); 0–14
Missing	7 (4·1)	25 (15·6)
Sex, *n* (%)		
Male	67 (39·4)	92 (57·5)
Female	102 (60·0)	67 (41·9)
Other	1 (0·6)	1 (0·6)
Ethnicity, *n* (%)		
White	136 (80·0)	95 (59·4)
Asian	21 (12·4)	33 (20·6)
Black, African, Caribbean	2 (1·2)	8 (5·0)
Arabic	2 (1·2)	1 (0·6)
Other	2 (1·2)	6 (3·8)
Mixed white/Asian	1 (0·6)	2 (1·3)
Mixed white/black	5 (2·9)	11 (6·9)
Mixed other	0 (0)	2 (1·3)
Prefer not to say	1 (0·6)	2 (1·3)
Socioeconomic group, *n* (%)		
I. Higher managerial, administrative, professional	16 (9·4)	23 (14·4)
II. Intermediate occupations	23 (13·5)	21 (13·1)
III. Small employers and own accounts	7 (4·1)	2 (1·3)
IV. Lower supervisory and technical occupations	23 (13·5)	22 (13·8)
V. Semiroutine and routine occupations	57 (33·5)	50 (31·3)
Unemployed	5 (2·9)	5 (3·1)
Other	29 (17·1)	27 (16·9)
Missing	10 (5·9)	10 (6·3)

Numbers of missing data are given only where data were missing.

**Table 3:** Diagnostic and quality‐of‐life characteristics of cases (psoriasis) and controls (other inflammatory skin diseases) included in the DIPSOC study

	Cases (*n* = 170)	Controls (*n* = 160)
**Diagnosis**, *n* (%)		
Psoriasis	170 (100)	NA
Eczema	NA	152 (95·0)
Ichthyosis	NA	3 (1·9)
Lichen planus	NA	2 (1·3)
Keratosis pilaris	NA	1 (0·6)
Viral exanthem	NA	1 (0·6)
Nonbullous congenital ichthyosiform erythroderma	NA	1 (0·6)
**Psoriatic arthritis**, *n* (%)	3 (1·8)	NA
**Histological diagnosis**, *n* (%)	4 (2·4)	4 (2·5)
**Consultation type**, *n* (%)		
New	59 (34·7)	48 (30·0)
Follow‐up	111 (65·3)	112 (70·0)
**Disease severity**, *n* (%)		
Mild or very mild	33 (19·4)	30 (18·8)
Moderate	32 (18·8)	33 (20·6)
Severe or very severe	22 (12·9)	31 (19·4)
Not documented	83 (48·8)	66 (41·3)
PASI, median (IQR)	4·9 (2·5–11·5)	NA
PASI missing	104 (61·2)	NA
**Quality of life**		
CDLQI score, mean (SD)	8·0 (6·2)	9·8 (5·8)
CHU9D utility score, median (IQR)	0·89 (0·13)	0·86 (0·18)
Not completed < 4 years old	3	52
Missing	0	2
**Current treatment**, *n* (%)		
Topical	158 (92·9)	150 (93·8)
Systemic	24 (14·1)	39 (24·4)
Phototherapy	10 (5·9)	4 (2·5)
**Diagnostic criteria assessor**, *n* (%)		
Dermatology consultant	4 (2·4)	3 (1·9)
Paediatric consultant	3 (1·8)	5 (3·1)
Dermatology registrar/fellow	54 (31·8)	39 (24·4)
Dermatology trained nurse	32 (18·8)	35 (21·9)
Other doctors	6 (3·5)	11 (6·9)
Other nurse	55 (32·4)	47 (29·4)
Other investigator	16 (9·4)	20 (12·5)

CDLQI, Children’s Dermatology Life Quality Index; CHU9D, Child Health Utility 9D; IQR, interquartile range; NA, not applicable; PASI, Psoriasis Area and Severity Index.

Nearly all of the controls were diagnosed with atopic dermatitis/eczema (referred to as eczema from here onwards) (94·4%). A small proportion of cases and controls had supporting histological diagnosis (< 3%). Where disease severity was documented, 12·9% of cases and 19·4% of controls had severe or very severe disease. For cases, the median Psoriasis Area and Severity Index was 4·9 (interquartile range 2·5–11·5). Mean CDLQI scores were similar between cases and controls (8·0 vs. 9·8). Approximately one‐third of the cases and controls were new consultations (34·7% vs. 30·0%) and around one‐fifth were receiving systemic treatment (14·1% vs. 24·4%) or phototherapy (5·9% vs. 2·5%).

### Objective 1: Diagnostic accuracy of consensus‐agreed criteria

**Figure 1:**
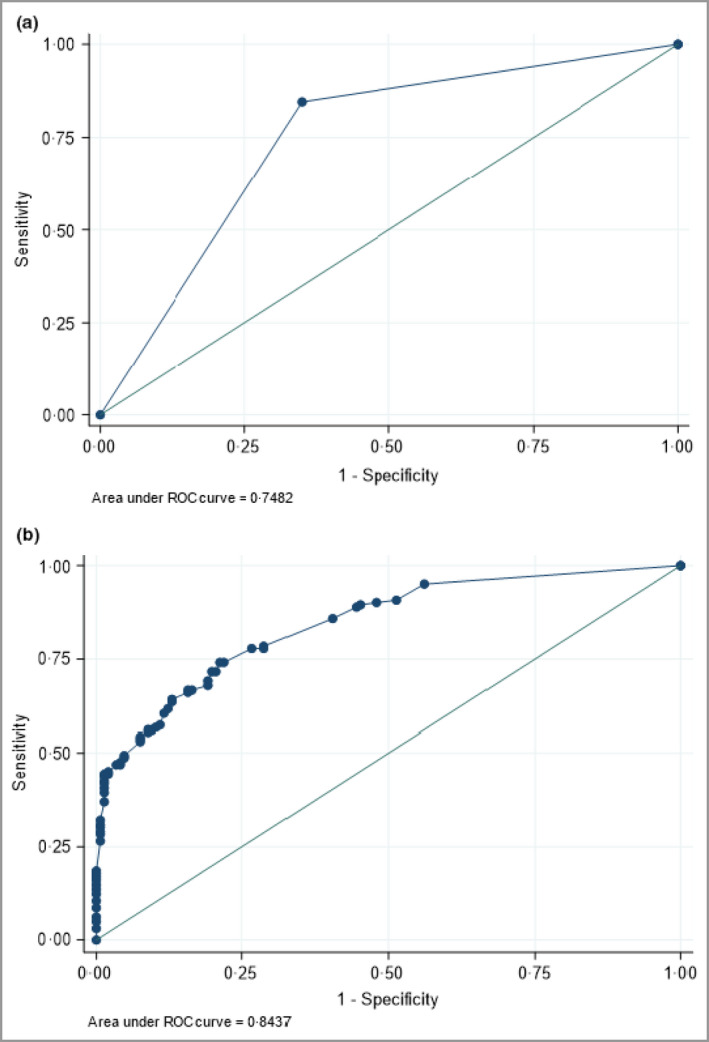
Receiver operator characteristic (ROC) curves for (a) the consensus‐agreed criteria and (b) the prediction model for the best predictive criteria. Complete‐case analysis, *n* = 308.

The frequency, univariate odds ratio, sensitivity, specificity and likelihood ratios of the individual diagnostic criteria are presented in Table 4. There were 16 consensus‐agreed criteria; the proposed threshold to support a diagnosis of psoriasis was one major and/or three or more minor criteria. The diagnostic accuracy and discrimination results for the consensus‐agreed criteria were 82·9% sensitivity, 65% specificity, AUC 0·74 [95% confidence interval (CI) 0·69–0·79], 2·37 positive likelihood ratio (+LR) and 0·26 negative likelihood ratio (−LR) (*n* = 320). The diagnostic accuracy results were similar for the complete‐case analysis (*n* = 308): 84·6% sensitivity, 65·1% specificity, AUC 0·75 (95% CI 0·70–0·80), +LR 2·42 and −LR 0·24 (Figure 1).

**Table 4:** Frequency, sensitivity, specificity, univariate odds ratio (OR) and likelihood ratios (LRs) of the 18 diagnostic criteria tested in the DIPSOC study

Diagnostic criteria	Cases, *N* = 170	Controls, *N* = 160	Univariate OR (95% CI)	Sensitivity (95% CI)	Specificity (95% CI)	+ve LR	−ve LR
	*n* (%); missing						
DC1. Scale and erythema in the scalp involving the hairline	89 (52·4)	27 (16·9)	5·41 (3·12–9·4)	52·4 (44·7–60·1)	83·1 (76·4–88·6)	3·10	0·57
DC2. Retroauricular erythema	85 (50·0)	34 (21·3)	3·71 (2·23–6·16)	50 (42·4–57·8)	78·8 (71·6–84·8)	2·36	0·63
DC3. Scaly erythema inside the external auditory meatus	77 (45·3)	19 (11·9); 1	6·1 (3·32–11·2)	45·3 (37·7–53·1)	88·1 (82–92·6)	3·81	0·62
DC4. Persistent well‐demarcated facial rash with fine or absent scale	55 (32·4)	22 (13·8)	3 (1·7–5·29)	32·4 (25·4–39·9)	86·3 (79·9–91·2)	2·36	0·78
DC5. Persistent well‐demarcated erythematous scaly rash anywhere on the body	119 (70·4); 1	39 (24·5); 1	7·32 (4·22–12·7)	70·4 (62·9–77·2)	75·5 (68–81·9)	2·87	0·39
DC6. Scaly erythematous plaques on the trunk triggered by a sore throat or other infection	19 (11·2)	4 (2·5); 1	4·03 (1·30–12·5)	9·4 (5·48–14·8)	97·5 (93·7–99·3)	3·76	0·93
DC7. Raindrop plaques typical of guttate disease on the trunk or limbs	50 (29·4)	12 (7·5)	5·10 (2·53–10·3)	29·4 (22·7–36·9)	92·5 (87·2–96·0)	3·92	0·76
DC8. Persistent erythema in the umbilicus	37 (21·8)	4 (2·5)	10·9 (3·59–32·8)	21·8 (15·8–28·7)	97·5 (93·8–99·3)	8·72	0·80
DC9. Scaly erythematous plaques on the extensor surfaces of the elbows and/or knees	88 (51·8)	35 (21·9)	3·83 (2·31–6·36)	51·8 (44–59·5)	78·1 (70·9–84·3)	2·37	0·62
DC10. Nail pitting	26 (15·7); 4	15 (9·4); 1	1·78 (0·90–3·52)	15·7 (10·5–22·1)	90·6 (84·9–94·6)	1·67	0·93
DC11. Onycholysis	9 (5·4); 3	6 (3·8); 1	1·45 (0·50–4·19)	5·4 (2·5–9·98)	96·2 (91·9–98·6)	1·42	0·98
DC12. Subungual hyperkeratosis	7 (4·2); 3	1 (0·6); 1	6·9 (0·83–57·8)	4·2 (1·7–8·4)	99·4 (96·5–99·9)	7	0·96
DC13. Fusiform swelling of a toe or finger	2 (1·2)	0; 1	–	1·2 (0·14–4·18)	100 (97·8–100)	–	0·99
DC14. Fine scaly patches involving the upper thighs and/or buttocks	74 (44·3); 3	34 (22·1); 6	2·81 (1·70–4·64)	44·3 (36·6–52·2)	77·9 (70·5–84·2)	2·00	0·72
DC15. Well‐demarcated erythematous rash in the napkin area involving the crural fold^a^	35 (20·7); 1	5 (3·2); 4	7·89 (2·89–21·5)	20·7 (14·9–27·6)	96·8 (92·7–98·9)	6·47	0·82
DC16. Natal cleft erythema and/or skin splitting	18 (10·7); 1	7 (4·4); 2	2·57 (1·04–6·38)	10·7 (6·44–16·3)	95·6 (91·1–98·2)	2·43	0·93
DC17. Koebner phenomenon	9 (5·3)	2 (1·3); 9	4·36 (0·92–20·8)	5·3 (2·45–9·81)	98·7 (95·5–99·8)	4·08	0·96
DC18. Family history – first and second degree	102 (60·0)	43 (26·9)	4·08 (2·49–6·69)	60 (52·2–67·4)	73·1 (65·6–79·8)	2·23	0·55
DC18a. Family history – first degree	64 (37·7)	15 (9·4)	5·84 (3·04–11·2)	37·7 (30·3–45·4)	90·6 (85–94·7)	4·01	0·69
DC18b. Family history – second degree	72 (42·4)	32 (20·0)	2·94 (1·77–4·89)	42·4 (34·7–50·2)	80 (73·0–85·9)	2·12	0·72

CI, confidence interval. ^a^Napkin area is used to describe an area of skin in children and young people of all ages. It refers to the area that would be covered by a nappy in younger children.

### Objective 1: Predictive model development and diagnostic accuracy

**Figure 2:**
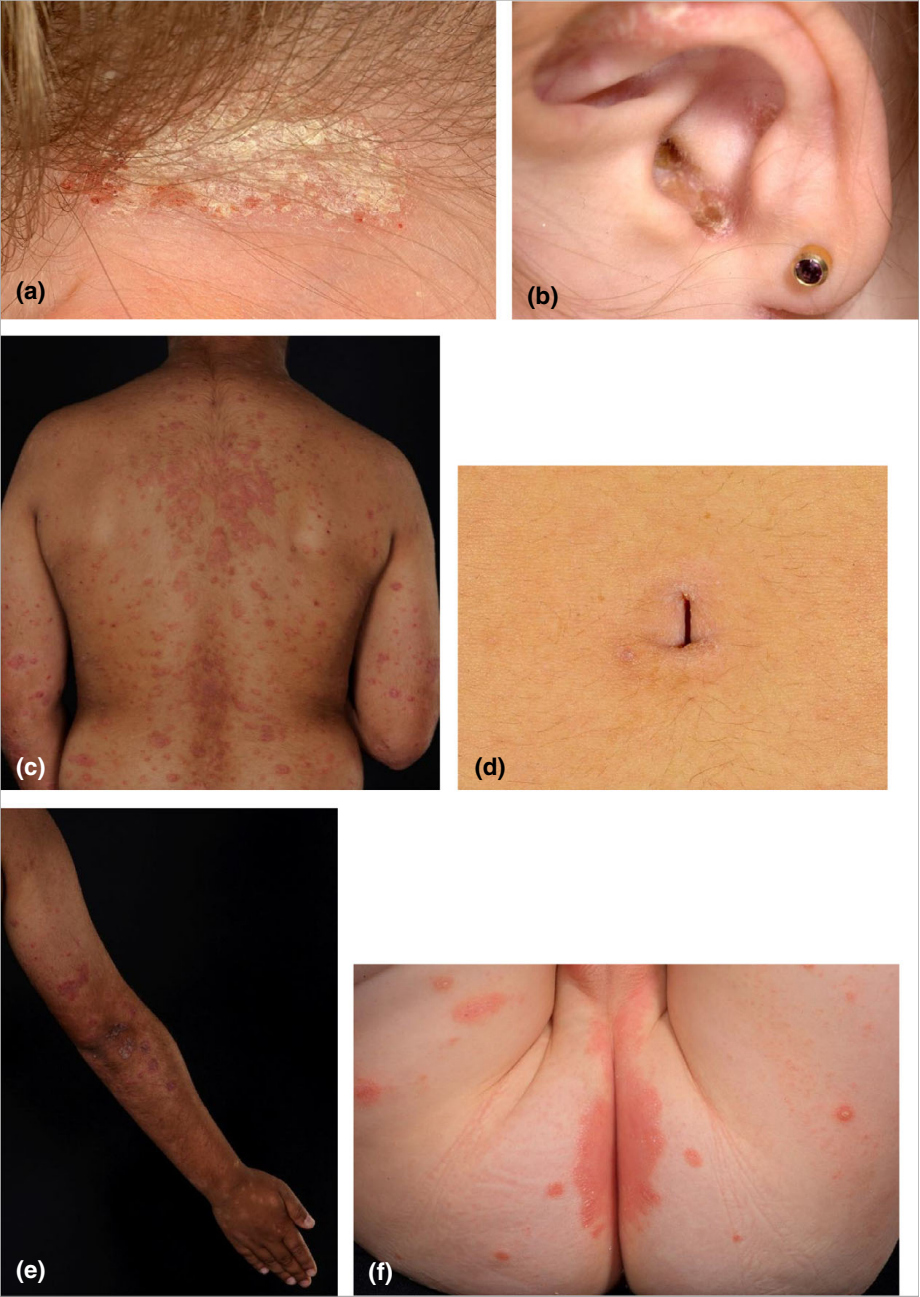
Photographs showing the six clinical signs of the best predictive criteria for psoriasis in children and young people. One image per criterion has been chosen and therefore will not be representative of all skin changes and skin tones in which psoriasis can be seen. (a) Scale and erythema in the scalp involving the hairline. (b) Scaly erythema inside the external auditory meatus. (c) Persistent well‐demarcated erythematous scaly rash anywhere on the body. (d) Persistent erythema in the umbilicus. (e) Scaly erythematous plaques on the extensor surfaces of the elbows and/or knees. (f) Well‐demarcated erythematous rash in the napkin area involving the crural fold.

In total 18 diagnostic criteria were evaluated (16 consensus agreed plus two borderline consensus criteria). Two diagnostic criteria (hyperkeratosis of the nails, fusiform swelling of a finger or toe) with fewer than 10 observations were excluded from the predictive model. None of the three consensus‐agreed major criteria reached the a priori threshold definition for major criteria. Therefore, all of the remaining 16 criteria were available for model selection. Seven criteria were retained in the logistic regression model and are referred to as the ‘best predictive criteria’ (Table 5, Figure 2).

**Table 5:** Adjusted multivariate odds ratios (ORs) and coefficient values of the seven best predictive diagnostic criteria in the prediction model

Diagnostic criteria	OR (95% CI)	Wald *P*‐value	Coefficient
DC1. Scale and erythema in the scalp involving the hairline	2·17 (1·06–4·44)	0·034	0·595
DC3. Scaly erythema inside the external auditory meatus	2·06 (0·95–4·44)	0·067	0·644
DC5. Persistent well‐demarcated erythematous scaly rash anywhere on the body	2·79 (1·46–5·32)	0·002	1·013
DC8. Persistent erythema in the umbilicus	3·06 (0·92–10·2)	0·068	1·173
DC9. Scaly erythematous plaques on the extensor surfaces of the elbows and/or knees	2·01 (1·06–3·82)	0·032	0·701
DC15. Well‐demarcated erythematous rash in the napkin area involving the crural fold	2·66 (0·85–8·30)	0·091	1·050
DC18. Family history	3·66 (2·05–6·54)	< 0·001	1·276

CI, confidence interval.

The diagnostic accuracy and discrimination results of the best predictive criteria based on the highest proportion correctly classified were sensitivity 76·8%, specificity 72·7% and AUC 0·84 (95% CI 0·79–0·88) (*n* = 308) (Figure 1). The Hosmer–Lemeshow statistic indicated good calibration (*P* = 0·66).

#### Internal validation of the predictive model

The *c*‐statistic in the original sample was 0·84 (95% CI 0·80–0·85), in the bootstrapped sample it was 0·85 (95% CI 0·78–0·91) and the average optimism of the *c*‐statistic was 0·012 (95% CI −0·06 to 0·07) (Table S1; see Supporting Information).

#### Diagnostic accuracy of the predictive model stratified for subgroups

Stratification to assess the diagnostic accuracy of the predictive model was possible for age at assessment, sex, and dermatological experience of the assessor, and shows reasonably comparable performance across the groups (Appendix S1; see Supporting Information). The stratification showed the performance to be comparable, suggesting the criteria are suitable to be used across different populations.

#### Missing data

The percentage of missing data for the presence or absence of the diagnostic criteria was < 7%. Criteria involving the nails (covered by nail varnish) or napkin area were more likely to be missing. The effect of missing data was explored and there was no substantial change in the diagnostic accuracy of the predictive model when all missing observations were coded as ‘yes’ (73·5% sensitivity, 74·4% specificity, AUC 0·83) or ‘no’ (72·4% sensitivity, 76·3% specificity, AUC 0·83) (*n* = 330).

#### Worked examples using the predictive criteria

The final equation for the prediction of psoriasis in children (< 18 years) is as follows.

$$\begin{array}{c} \text{Probability}\;\text{of}\;\text{psoriasis}\;=\;\exp(-1.717\;+\;0.595\times \text{DC}1\;+\;0.644\times \text{DC}3\;+\;1.013\times \text{DC}5\;+\;\\ 1.173\times \text{DC}8\;+\;0.701\times \text{DC}9\;+\;1.05\times \text{DC}15\;+\;1.276\times \text{DC}18)/[1\;+\;\exp(-1.717\;+\;\\ 0.595\times \text{DC}1\;+\;0.644\times \text{DC}3\;+\;1.013\times \text{DC}5\;+\;1.173\times \text{DC}8\;+\;0.701\times \text{DC}9\;+\;1.05\times \text{DC}15\;+\;\\ 1.276\times \text{DC}18)]. \end{array}$$

A score of 1 is used if a criterion is present (positive) and a score of 0 if a criterion is absent (negative). This equation can be used to calculate the probability that a child has psoriasis. Worked examples are provided in Appendix S1.

#### Diagnostic accuracy of the best predictive criteria

Table 6 provides data on the diagnostic accuracy of different numbers of positive diagnostic criteria. These results suggest that the presence of two or more diagnostic criteria can correctly identify 78·4% of children with psoriasis (sensitivity or the true‐positive rate), and 28·8% of children without psoriasis will be incorrectly identified as having psoriasis (1 − specificity or the false‐positive rate). These are the closest values to the prespecified threshold of 80% sensitivity and 80% specificity.

**Table 6:** Frequency, sensitivity and specificity of different numbers of best predictive positive diagnostic criteria

Number of diagnostic criteria	Cases, *n* (%)		Controls, *n* (%)	
	*N* = 162	Sensitivity	*N* = 146	Specificity
1 or more	154 (95·1)	95·1%	82 (56·2)	43·8%
2 or more	127 (78·4)	78·4%	42 (28·8)	71·2%
3 or more	104 (64·2)	64·2%	19 (13·0)	87·0%
4 or more	75 (46·3)	46·3%	6 (4·1)	95·9%
5 or more	47 (29·0)	29·0%	1 (0·7)	99·3%
6 or more	17 (10·5)	10·5%	0	0
7	5 (3·1)	3·1%	0	0

### Objective 2: Comparing the consensus‐agreed criteria and the best predictive criteria

The ROC curves for the two sets of criteria are presented in Figure 1.

### Objective 2: Interobserver variability

The kappa statistics comparing assessment 1 and assessment 2 for each of the 18 diagnostic criteria for the first 40 participants recruited to the DIPSOC study are presented in Table S2 (see Supporting Information).

## Discussion

The consensus‐agreed diagnostic criteria achieved good diagnostic accuracy using the expert‐agreed cutoff of one major or at least three minor criteria. The consensus criteria were found to have higher sensitivity than specificity, and the AUC showed that discrimination between cases and controls was moderate (AUC 0·74).^22^ Refinement of the criteria into a shorter list of seven ‘best predictive’ criteria was achieved using multivariate analysis (Figure 2): (i) scale and erythema in the scalp involving the hairline, (ii) scaly erythema inside the external auditory meatus, (iii) persistent well‐demarcated erythematous rash anywhere on the body, (iv) persistent erythema in the umbilicus, (v) scaly erythematous plaques on the extensor surfaces of the elbows and/or knees, (vi) well‐demarcated erythematous rash in the napkin area involving the crural fold and (vii) family history of psoriasis. Three of these criteria involve skin in hidden sites, which are often covered by clothing or hair.

The diagnostic accuracy of the predictive model was also good (sensitivity 76·8%, specificity 72·7%), with a slightly higher AUC (0·84). The model nearly reached the desired diagnostic accuracy of 80% sensitivity and 80% specificity. After applying different cutoffs for the number of best predictive criteria, two or more criteria is a proposed scoring cutoff, which gives 78·4% sensitivity and 71·2% specificity. The criteria performed sufficiently similarly in younger and older children and when assessed by those with and without dermatology training. There was a difference in age at onset of symptoms between children with and without psoriasis, which would be interesting to explore as a criterion (predictor) in future studies.

Validated clinical diagnostic criteria for different skin diseases are very few in number. Most studies have developed multiple sets of diagnostic criteria for two diseases: eczema and Behçet disease.^23^, ^24^ This is evidence that research to develop diagnostic criteria has not been prioritized for skin disease. This deficit is being addressed for psoriasis in adults through research coordinated by the Global Psoriasis Atlas (www.globalpsoriasisatlas.org). A consensus study with psoriasis experts has identified nine criteria to support the diagnosis of chronic plaque psoriasis in adults, focusing on the clinical appearance of skin lesions.^25^ Diagnostic accuracy and validation studies for these criteria are now needed.

The DIPSOC study has been designed with careful adherence to key quality components in diagnostic accuracy studies.^19^, ^26^, ^27^ Consecutive patients were approached and the exclusion criteria kept to a minimum to minimize selection bias. Bias related to the index test was minimized through blinded assessments and prespecifying the diagnostic threshold. The DIPSOC study recruited from 12 UK paediatric dermatology departments, which provides clinical diversity of patients and broader representation of a dermatologist’s diagnosis than a single‐centre study. The study recruitment target was successfully reached. The diagnostic accuracy of the model was explored for different populations and clinical settings, which are important for the clinical application of the criteria. Investigators received standardized training but had a range of dermatological experience; this better reflects the broad final use of the diagnostic criteria.

An important limitation of the study is the choice of study design and setting. A case–control design was chosen as a feasible study design to test the diagnostic accuracy of the consensus‐agreed criteria and provide sufficient data for the prediction model. The study design and recruitment from secondary care are likely to have introduced both selection and spectrum bias, which lead to overestimation of the diagnostic accuracy.^26^ However, the decision to include controls with skin disease instead of healthy controls will have minimized this bias.^28^ Nearly all controls had a diagnosis of eczema, and therefore the discriminatory ability of the criteria may be different when comparing against a more diverse group of controls. Using a case–control design also fixes the prevalence, therefore it is not possible to calculate the positive and negative predictive values. It was not possible to explore the variability in the reference standard as planned due to insufficient clinical images of suitable quality. All participants were required to have a dermatologist’s diagnosis made in a paediatric dermatology clinic, but no data were collected on the experience or paediatric dermatology training of the clinician.

The DIPSOC study was a development study and is the starting point for further testing and potential evolution of the diagnostic criteria. Future research should include validation of the criteria in an external cohort. Complementary studies could identify a shortlist of criteria using alternative techniques such as decision making based on motivated choice or latent class analysis.

The coefficient values from the prediction model can be used as per the worked examples to calculate the probability of a child developing psoriasis. However, the formula is unlikely to be used in routine clinical practice, and further scoping work is needed to establish whether there is appetite for an accessible risk calculator. The sensitivity and specificity of the predictive model are also not directly applicable to a clinical or research population, because this is the diagnostic accuracy of the model performance and not a specific number of criteria. Therefore, to provide a more intuitive way for using the criteria, external cutoffs in the number of criteria were explored.

It is estimated that if any two of the seven criteria are present, this will identify psoriasis in 78% of children with psoriasis (sensitivity) and rule out psoriasis with 71% certainty in children with other skin disorders (specificity). The acceptability of these values for clinical practice will need to be explored with clinicians. Depending on the setting and purpose of using the diagnostic criteria, the number of criteria required to support a diagnosis of psoriasis could be decreased or increased, to improve sensitivity and specificity, respectively. For example, for recruitment into clinical trials a higher specificity would be desirable. Increasing the cutoff to any four of the seven criteria increases the specificity to 96%.

In conclusion, this study provides history‐ and examination‐based data on the clinical features of psoriasis in children and proposes seven diagnostic criteria with good discriminatory ability in secondary‐care patients. Three of the best predictive criteria involve skin in hidden sites, such as umbilicus, groin flexures and external auditory meatus. These criteria will therefore be helpful to prompt examination of these specific areas to determine whether a patient has psoriasis or not. The DIPSOC study was designed as a development study and is a promising first step. Further studies are planned to explore and validate the diagnostic performance of individual criteria and the collective seven best predictive criteria in different datasets and settings.

## Author Contribution

**Esther Burden‐Teh:** Conceptualization (equal); Formal analysis (equal); Funding acquisition (lead); Investigation (lead); Methodology (equal); Project administration (lead); Writing‐original draft (lead); Writing‐review & editing (lead). **Ruth Murphy:** Conceptualization (equal); Formal analysis (supporting); Funding acquisition (equal); Investigation (equal); Methodology (equal); Supervision (equal); Writing‐original draft (supporting); Writing‐review & editing (equal). **Sonia Gran:** Conceptualization (supporting); Formal analysis (equal); Funding acquisition (supporting); Investigation (supporting); Methodology (equal); Supervision (equal); Writing‐original draft (supporting); Writing‐review & editing (equal). **Tamar Nijsten:** Conceptualization (supporting); Formal analysis (equal); Methodology (equal); Writing‐original draft (supporting); Writing‐review & editing (equal). **Carolyn Hughes:** Conceptualization (equal); Formal analysis (supporting); Investigation (supporting); Methodology (equal); Writing‐original draft (supporting); Writing‐review & editing (equal). **Alya Abdul‐Wahab:** Investigation (equal); Writing‐review & editing (equal). **Anthony P Bewley:** Investigation (equal); Writing‐review & editing (equal). **Nigel Burrows:** Investigation (equal); Writing‐review & editing (equal). **Sharmela. Darne:** Investigation (equal); Methodology (supporting); Writing‐review & editing (equal). **Joanna Gach:** Investigation (equal); Writing‐review & editing (equal). **Ru Katugampola:** Investigation (equal); Writing‐review & editing (equal). **catherine Jury:** Investigation (equal); Writing‐review & editing (equal). **Kar‐Hung Kuet:** Investigation (equal); Writing‐review & editing (equal). **Joanne Llewellyn:** Investigation (equal); Methodology (supporting); Writing‐review & editing (equal). **Tess McPherson:** Investigation (equal); Writing‐review & editing (equal). **Jane Ravenscroft:** Investigation (equal); Writing‐review & editing (equal). **Saleem Taibjee:** Investigation (equal); Methodology (supporting); Writing‐review & editing (equal). **Cairine Wilkinson:** Investigation (equal); Writing‐review & editing (equal). **Kim S Thomas:** Conceptualization (equal); Formal analysis (equal); Funding acquisition (equal); Investigation (equal); Methodology (equal); Supervision (lead); Writing‐original draft (supporting); Writing‐review & editing (equal).

## Supporting information


**Appendix S1** Supplementary methods.
**Table S1** Internal validation of the predictive model.
**Table S2** Interobserver variability.
