# Supplementary material for: Identifying the best predictive diagnostic criteria for psoriasis in children (< 18 years): a UK multicentre case–control diagnostic accuracy study (DIPSOC study)
Source: Br J Dermatol. 2021 Nov 24;186(2):341–51. doi: 10.1111/bjd.20689 (PMC9298773; doi:10.1111/bjd.20689)
Supplement: Supplementary file 1 — Appendix S1 Supplementary methods. Table S1 Internal validation of the predictive model. Table S2 Interobserver variability. [file BJD-186-341-s001.docx]

DIPSOC Supplementary 1

*Diagnostic accuracy of the predictive model stratified for subgroups*

The sensitivity, specificity and AUC for <10 years vs ≥10 years were as follows: 65.7% vs 85.3%, 89.8% vs 76.6%, 0.83 (0.77, 0.89) vs 0.87 (0.81, 0.93). The sensitivity, specificity and AUC for males vs females were as follows: 64.2% vs 76.1%, 89.5% vs 74.6%, 0.86 (0.80, 0.92) vs 0.82 (0.75, 0.89). The sensitivity, specificity and AUC for dermatology background vs no dermatology background were as follows: 78.9% vs 66.7%, 77.3% vs 87.3%, 0.85 (0.79, 0.90 vs 0.84 (0.78, 0.90).

*Worked examples using the predictive criteria*

A child with all 7 best predictive criteria has a 99% probability of having psoriasis. Linear predictor = -1.717 + 0.595(1) + 0.644(1) + 1.013(1) + 1.173(1) + 0.701(1) + 1.05(1) + 1.276(1)=4.735. Probability of having psoriasis =exp(4.375)/(1+ exp(4.375))=0.99.

A child with the following 2 diagnostic criteria “scale and erythema on the scalp involving the hairline” and “positive family history” has a 54% probability of psoriasis. Linear predictor =-1.717 + 0.595(1) + 1.276(1)=0.154. Probability of having psoriasis= exp(0.154)/(1+ exp(0.154))=0.54.

**Table S1** Internal validation of the predictive model

|  | **Mean** | **95%CI** |
| --- | --- | --- |
| **Apparent performance** |  |  |
| c-statistic | 0.85 | 0.78, 0.91 |
| Calibration in the large (CITL) | 2.24 | -6.22, 1.62 |
| Calibration slope | 1 | 1, 1 |
|  |  |  |
| **Original sample** |  |  |
| c-statistic | 0.84 | 0.80, 0.85 |
| Calibration in the large (CITL) | 0.02 | -0.36, 0.45 |
| Calibration slope | 0.93 | 0.67, 1.17 |
|  |  |  |
| **Optimism in the performance** |  |  |
| C-statistic | 0.01 | -0.06, 0.07 |
| Calibration in the large (CITL) | -0.02 | -0.45, 0.36 |
| Calibration slope | 0.07 | -0.17, 0.33 |
|  |  |  |

**Table S2** Interobserver variability

| **Diagnostic criteria** | **Kappa*** | **% agreement** |
| --- | --- | --- |
| DC1* | 0.69 | 84.6 |
| DC2 | 0.49 | 74.4 |
| DC3* | 0.68 | 84.6 |
| DC4 | 0.56 | 81.1 |
| DC5* | 0.64 | 82.1 |
| DC6 | 0.45 | 89.7 |
| DC7 | 0.59 | 84.2 |
| DC8* | 0.62 | 92.3 |
| DC9* | 0.38 | 69.2 |
| DC10 | 0.21 | 79 |
| DC11 | 0.23 | 87.2 |
| DC12 | 0.65 | 97.5 |
| DC13 | 0 | 97.4 |
| DC14 | 0.47 | 77.1 |
| DC15* | 0.59 | 86.5 |
| DC16 | 0.54 | 89.2 |
| DC17 | -0.0354 | 92.3 |
| DC18a* | 1 | 64.5 |
| DC18b* | 1 | 57.4 |

*One of the seven best predictive criteria. 18a and 18b were combined as one diagnostic criterion “family history”.
